# Immunoregulatory Enzymes

**DOI:** 10.32607/actanaturae.27549

**Published:** 2025

**Authors:** S. V. Kupriyanov, K. M. Kirilenko, D. N. Starkov

**Affiliations:** Tomsk State University, Laboratory of Evolutionary Cytogenetics, Tomsk, 634050 Russian Federation

**Keywords:** Immunometabolism, immune response regulation, enzymatic regulation

## Abstract

Immunoregulatory enzymes, which function both as biological catalysts and regulatory elements, play a crucial role in controlling immune responses. Dysfunction of these proteins can contribute to various pathological conditions, such as the suppression of antitumor immunity or impairment of anti-infectious immune responses. This review discusses the most extensively studied immunoregulatory enzymes, including indoleamine 2,3-dioxygenase 1, arginase 1, inducible nitric oxide synthase, glyceraldehyde-3-phosphate dehydrogenase, and ectonucleoside triphosphate diphosphohydrolase 1. Their classification is provided, along with an analysis of the distinctive characteristics inherent to this group of enzymes. Additionally, new directions for the medical application of immunoregulatory enzymes are explored.

## INTRODUCTION

The primary function of the immune system is to maintain homeostasis by eliminating foreign agents, such as pathogens, as well as aberrant self-cells [1]. This applies not only to tumor cells, but also to immune cells, whose uncontrolled activity can be detrimental to the host, leading to autoimmune or allergic disorders. Therefore, regulation of the immune system can be regarded as a central mechanism that ensures its proper function.

The metabolism of immune cells differs significantly from that of other systems in the body. Many specialized immune functions, such as proliferation in response to antigen stimulation or the synthesis and release of cytotoxic agents for pathogen defense, necessitate metabolic reprogramming [2]. A key example is the Warburg effect, which is a prerequisite for the activation of many lymphocyte types. This phenomenon is characterized by the diversion of pyruvate, generated through glycolysis, away from the pyruvate dehydrogenase complex toward lactate production, despite the absence of hypoxia, distinguishing it from anaerobic glycolysis [3]. The field of immunometabolism investigates the metabolic processes involved in immune responses [4], with one of its key aspects being the regulation of immune function via metabolic pathways. A crucial role in this regulation is played by immunoregulatory enzymes. However, there is currently no universally accepted definition of what constitutes an immunoregulatory enzyme. Instead, several representative enzymes have been identified, including indoleamine 2,3-dioxygenase 1 (IDO1) [5], arginase 1 (ARG1) [6], and glyceraldehyde- 3-phosphate dehydrogenase (GAPDH) [7], among others. The aim of this review is to systematize current knowledge on immunoregulatory enzymes.

It is important to emphasize that metabolic regulation of immune processes occurs not only at the level of individual enzymes, but also at the level of entire metabolic pathways [8, 9]. Glycolysis is a crucial process governing T-lymphocyte activation; however, its execution requires the coordinated activity of multiple enzymes. In this review, we do not classify such enzymes as immunoregulatory, since they function as components of a regulatory metabolic pathway. In contrast, expression of a single enzyme, such as IDO1, is sufficient to alter how the immune system functions [5], and this enzyme acts as an independent regulatory element. The review focuses on the enzymes that function in such a manner. Since research into immunoregulatory enzymes is still in its early stages, it is necessary to first identify the enzymes with known immunoregulatory properties and subsequently establish a definition for this class of enzymes as a whole. This work examines indoleamine 2,3-dioxygenase 1, arginase 1, inducible nitric oxide synthase, glyceraldehyde-3-phosphate dehydrogenase, and ectonucleoside triphosphate diphosphohydrolase 1, since these enzymes represent the most extensively studied members of the immunoregulatory enzyme group and exemplify key regulatory mechanisms. Based on the properties of these proteins, we propose a classification of immunoregulatory enzymes according to their mechanism of action and site of activity.

## IDO1

Indoleamine 2,3-dioxygenase 1 (IDO1) is an enzyme involved in tryptophan catabolism [10], although its substrate specificity is not strictly confined to tryptophan. The immunosuppressive effect of IDO1 is primarily associated with the conversion of tryptophan to kynurenines. IDO1 is expressed by antigen-presenting cells [11] and is strongly induced by interferon- gamma (IFN-γ) [12]. Notably, the immunosuppressive activity of IDO1 is most prominent against T helper type 1 (Th1) cells [13], which *per se *produce IFN-γ [14]. This creates a potential negative feedback loop limiting excessive proliferation of Th1 cells, thereby maintaining immune homeostasis.

– tryptophan depletion [15];

– production of kynurenines, which act through the aryl hydrocarbon receptor (AhR) [16];

– the non-enzymatic function as a signaling protein [17].

IDO1-mediated immunosuppression supports immunological tolerance in immune-privileged organs, such as the placenta [18] and the cornea [19].

A number of pathological conditions are associated with the dysfunction of the IDO1 system. For instance, the expression of this enzyme in tumors enables immune evasion, thereby promoting disease progression [20]. Certain pathogens have also evolved mechanisms to exploit IDO1 for host immune suppression. For example, *Leishmania major *and *L. donovani* can induce IDO1 expression in human dendritic cells, leading to the inhibition of lymphocyte proliferation and disruption of the immune response [21]. On the other hand, IDO1 has been shown to exert antibacterial effects against certain pathogens by depleting an essential substrate, tryptophan [22]. IDO1 inhibitors have been extensively studied as antitumor agents; however, their clinical efficacy remains limited despite promising preclinical results. This limitation may be due to the activation of alternative immunosuppressive mechanisms [23].

## ARG1

Arginase 1 (ARG1) catalyzes the conversion of arginine to ornithine and urea [24]. This enzyme prforms a regulatory activity through arginine depletion, since arginine is an essential amino acid for immune cells [25]. T-cell activation and differentiation are suppressed in an environment with active arginase and arginine deficiency; however, this mechanism is ineffective when arginine is abundant [26]. Murine models have demonstrated that in response to cytokine production by Th2 cells, macrophages express arginase, which regulates Th2 cell numbers and the inflammation induced by this cell population [27]. In humans, ARG1 expression by immune cells is also implicated in immune response regulation. Neutrophils isolated from the blood of septic patients were shown to suppress CD8^+^ T-lymphocyte proliferation in co-culture experiments due to ARG1 expression [28]. Similarly, neutrophils circulating in the blood of glioblastoma patients can degranulate arginase, thereby suppressing the activity of adaptive immune cells [29]. Notably, under normal conditions, neutrophils contain a high quantity of arginase-rich granules. Yet the enzyme does not interact with cytoplasmic arginine. As a result, neutrophil circulation does not lead to increased arginine consumption by the blood [30]. This suggests that degranulation may be necessary for activating the regulatory function of arginase. Other leukocytes within the peripheral blood mononuclear cell (PBMC) fraction have also been shown to express ARG1 in response to damaging factors [31], although it remains unclear whether this represents a regulatory mechanism. Notably, ARG1 also exhibits an antimicrobial activity. In human neutrophils, the enzyme is localized within specific granules and is released into the phagolysosome upon pathogen phagocytosis, leading to localized arginine depletion and subsequent microbial death [30]. The activity of macrophage arginase at the sites of specific inflammation may also help curb the spread of a pathogen, as demonstrated in murine models of the tuberculosis infection [32]. This mechanism is most likely to be associated with arginine depletion, since no direct effect of ARG1 metabolites on mycobacterial growth has been identified.

## iNOS

Unlike arginase and IDO1, inducible nitric oxide synthase (iNOS) functions as an immunoregulatory enzyme primarily within the innate immune system. Specifically, nitric oxide (NO) can suppress interleukin- 12 (IL-12) production in macrophages and dendritic cells, as demonstrated in animal models [33]. Additionally, NO acts as an antimicrobial agent [34], targeting intracellular pathogens. Its bactericidal effect is attributed to the formation of peroxynitrite, a potent oxidant that damages various cellular structures of the pathogen. Due to its short half-life, nitric oxide exerts its primary regulatory effects within NO-producing cells, where it nitrosylates functional amino acid residues such as tyrosine, in signaling proteins. Through nitrosylation, NO was shown to inhibit Th17 cell differentiation in mice [35], as well as M1 macrophage differentiation [36]. Since these studies were conducted in murine models, further investigation is required to assess their applicability to human cells. The expression of iNOS in innate immune cells regulates the production of proinflammatory cytokines, which contrasts with its role as a bactericidal agent. By analogy with ARG1, a hypothesis can be put forward that the subcellular localization of iNOS can be linked to the dual functionality of this enzyme.

## GADPH

Glyceraldehyde-3-phosphate dehydrogenase (GAPDH) is a key enzyme in glycolysis, the central pathway of glucose metabolism in immune cells [37]. Recently, a mechanism for immune response regulation in T-lymphocytes involving GAPDH has been described [38]. Under glucose-sufficient conditions, this enzyme facilitates glycolysis, which is essential for energy production and the supply of substrates for anabolic processes. However, under glucose-limiting conditions, GAPDH shifts to a regulatory function by recognizing specific motifs in certain mRNAs and promoting their degradation. This leads to a decrease in the expression of several proteins, including IFN-γ, the key cytokine of Th1 cells. As a result, T-lymphocytes are unable to synthesize IFN-γ in a glucose-deficient environment. This phenomenon may partially explain the reduced Th1 immune response activity observed in some tumor tissues, which also exhibit high glucose consumption. Indeed, glucose deprivation has been identified as an immunosuppressive factor within the tumor microenvironment [39]. Notably, cytokine production regulated by GAPDH can be subject to negative feedback, designed to limit excessive IFN-γ production during uncontrolled T-lymphocyte expansion. This mechanism prevents excessive glucose consumption by proliferating lymphocytes and helps maintain a metabolic balance in the immune response [40].

## ENTPD1

The enzyme ectonucleoside triphosphate diphosphohydrolase 1 (ENTPD1) is an exonucleotide phosphatase that hydrolyzes nucleotides to nucleosides [41]. ENTPD1, also known as CD39, is expressed on the surface of immune cells. The immunoregulatory function of CD39 is based on the breakdown of extracellular ATP into adenosine, which suppresses the activation of various immune cells, particularly macrophages and T-lymphocytes, through A2A receptors and their associated intracellular signaling pathways [42, 43]. This mechanism has been studied both in murine models and in human cells [44]. A substantial body of research, conducted in both animal models and patient-derived samples, indicates the involvement of ENTPD1 in immunosuppression across various oncological diseases [45]. Additionally, the hydrolysis of ATP in plasma by ENTPD1 localized on the surface of plasma cells is considered one of the mechanisms contributing to immunosuppression in patients who have experienced sepsis [46].

## CLASSIFICATION AND GENERAL CHARACTERISTICS OF IMMUNOREGULATORY ENZYMES

**Fig. 1:**
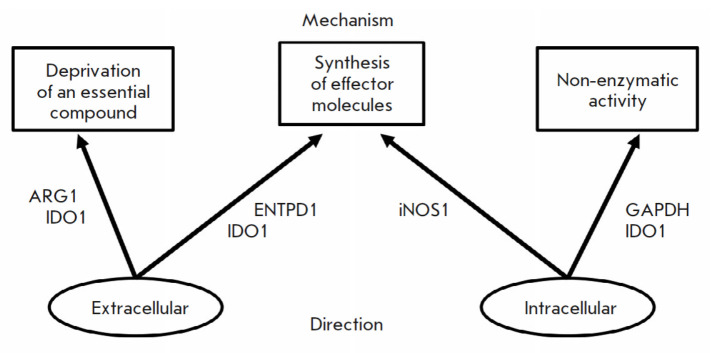
Classification of immunoregulatory enzymes

A classification can be established based on the available data on the described members of the immunoregulatory enzyme group (*Fig. 1*). Additionally, several common features of these enzymes can be identified, which may aid in the discovery of new members of this group.

**Classification**

Based on their mechanism of action, these enzymes can be classified into the following groups:

**Fig. 2:**
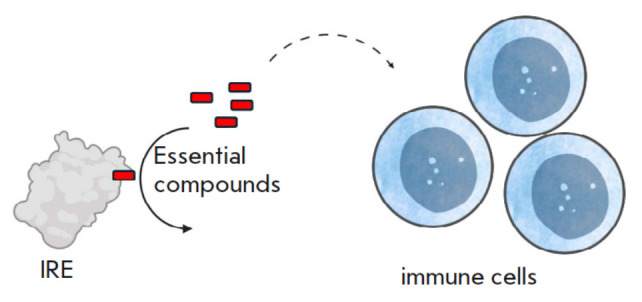
The regulation mechanism through the deprivation of essential and conditionally essential compounds

– enzymes mediating the deprivation of essential and conditionally essential compounds (*Fig. 2*);

**Fig. 3:**
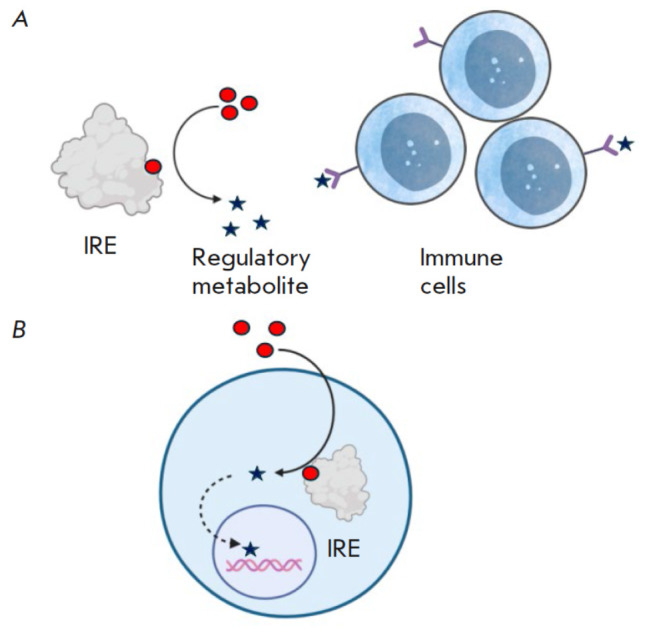
The mechanism of immune cell regulation through the synthesis of a regulatory metabolite. (A) Synthesis of a regulatory metabolite outside the cell (external regulation); (B) synthesis of a regulatory metabolite inside the cell (internal regulation)

– enzymes synthesizing a regulatory metabolite (*Fig. 3*); and

**Fig. 4:**
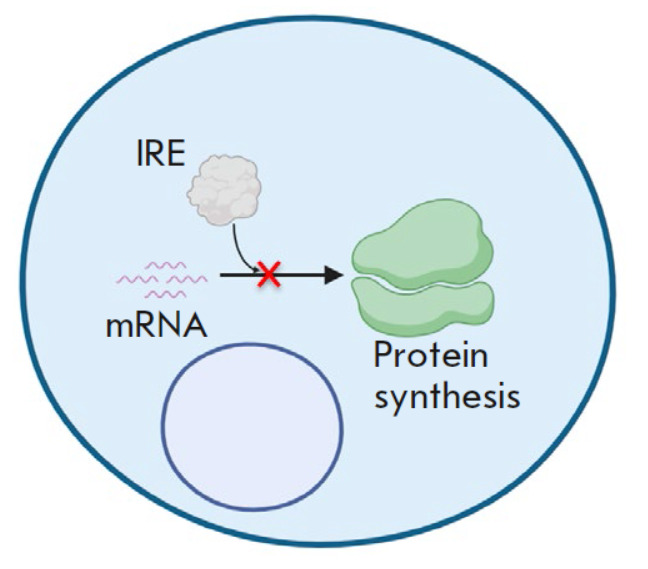
The mechanism of regulation through non-enzymatic activity

– enzymes exhibiting a non-enzymatic activity (*Fig. 4*).

The deprivation of essential compounds restricts the proliferative activity of cells; therefore, this strategy is primarily utilized in the regulation of the adaptive immune response, given the high proliferative activity of lymphocytes. This effect has a lesser impact on the populations of resting cells, whose metabolism is less intensive. Additionally, its effectiveness depends on the concentration of the essential compound, the tissue’s ability to synthesize or transport it, and external supplementation. For example, in a murine model of a *L. major *infection, the inhibitory effect of arginase on T-lymphocytes was neutralized by arginine administration [26]. Among the enzymes discussed, IDO1 (when functioning enzymatically) and ARG1 operate via this mechanism.

Regulation through the synthesis of regulatory metabolites, in contrast, does not affect all cells with a specific metabolic level in the microenvironment but rather targets specific populations expressing the corresponding receptors. This regulation can be either external or internal, depending on the localization of the enzymes and receptors for the regulatory metabolites. This mechanism is characteristic of IDO1, ENTPD1, and iNOS, with the action of NO being primarily confined to the producing cell, due to its rapid degradation.

Non-enzymatic activity implies that an enzyme possesses additional properties, such as the ability to influence intracellular signaling proteins or regulate mRNA levels. IDO1 and GAPDH exhibit this type of mechanism. The example of IDO1 highlights the fact that enzymes can simultaneously employ multiple regulatory mechanisms. For instance, iNOS has a potential to deplete arginine, an essential substrate for immune cells. However, there is currently no direct evidence confirming arginine deprivation by iNOS. In contrast, arginase, which also depletes the same substrate, is more efficient than iNOS, because its catalytic activity does not require oxygen, whose levels are often reduced in inflammatory foci [47].

Based on their direction, enzymes can be classified into the following groups:

– enzymes with an extracellular direction, and

– enzymes with an intracellular direction.

GAPDH, iNOS, and IDO1 (when IDO1 functions as a signaling protein) fall into intracellular direction, since they reside within cells and primarily influence gene expression in the cells where they are present. Notably, the intracellular activity of these proteins depends on the substrate levels in the cellular microenvironment, allowing for fine-tuned regulation of enzyme activity. This principle is best illustrated by GAPDH, which functions as a regulatory molecule only under conditions where its enzymatic activity is inhibited, such as in glucose deficiency.

Enzymes with extracellular direction, such as ARG1, ENTPD1, and IDO1 (when IDO1 functions enzymatically), influence not only the cells expressing them, but also surrounding cell populations. In some cases, these enzymes may not affect the cells in which they are expressed. For example, ENTPD1 expression appears to have no impact on plasmablasts, despite being localized on their surface [46].

**General characteristics of the function of immunoregulatory enzymes**

Enzymes involved in immune regulation share several characteristics; the most fundamental ones are their activation in response to immune system stimulation. The expression of these enzymes is dependent on immune response activators, such as pathogen-associated molecular patterns (PAMPs) and pro- or anti- inflammatory cytokines, as observed for IDO1 [12], ARG1 and iNOS [6], and ENTPD1 [48]. One exception to this pattern may be GAPDH; however, its regulatory activity is linked to the degradation of IFN-γ mRNA, whose expression is upregulated in response to PAMPs and cytokines [49]. As a consequence of this property, immunoregulatory enzymes are subject to negative feedback regulation. Upon activation by immune response stimuli (such as PAMPs and cytokines), they contribute to immune suppression and the maintenance of homeostasis. This mechanism prevents immune overactivation, which could otherwise lead to tissue damage and self-destruction [50].

The second key feature is the dependence of enzyme activity on the metabolic context in which it operates. The effects of deprivation-based enzymes can be neutralized if a sufficient substrate concentration is maintained. Conversely, the activity of enzymes producing regulatory metabolites is enhanced under conditions of substrate abundance, and diminished when substrate availability declines. While deprivation enzymes also lose their level of activity when substrate levels decrease, their regulatory effect is actually amplified, as their primary function — substrate depletion — is achieved. IDO1 represents a distinct case, since it functions under both substrate excess and deficiency. A hypothesis suggests that IDO1 preferentially suppresses Th1 cells over Th2 cells, as kynurenines exert a pro-apoptotic effect on Th1 cells, whereas tryptophan depletion merely arrests Th2 cell proliferation [13]. Hence, the action of IDO1 may also be context- dependent: an excess of tryptophan suppresses Th1 cells via kynurenine production, while tryptophan depletion leads to broader suppression, affecting Th2 cells through enhanced deprivation. Given that Th1 and Th2 cells exert mutually inhibitory effects [51], it can be hypothesized that under normal tryptophan concentrations, IDO1 supports a Th2-mediated immune response, whereas the overall T-cell activity is suppressed under conditions of tryptophan deficiency

Antimicrobial activity is another characteristic feature of some immunoregulatory enzymes (although not all of them are being discussed in this review). IDO1 [22], ARG1 [30], and iNOS [34] exhibit antimicrobial properties and are utilized by the immune system to combat specific pathogens. The mechanisms underlying the antimicrobial activity of these enzymes are analogous to their immunoregulatory functions: either through deprivation of essential compounds, thereby restricting the proliferative activity of the pathogen [22], or through the synthesis of antimicrobial metabolites [34]. It can be hypothesized that the original function of these enzymes was primarily to combat infectious agents, but they have also acquired a regulatory role over the course of evolution. This adaptation was likely to occur, because the metabolism of highly active immune cells, such as proliferating lymphocytes, resembles that of rapidly dividing pathogen cells (e.g., bacteria, fungi, and protozoa). It has been suggested that certain immunoregulatory mechanisms may have evolved from effector mechanisms originally designed for pathogen elimination.

## POTENTIAL IMMUNOREGULATORY ENZYMES AND STRATEGIES FOR THEIR IDENTIFICATION

Based on the characteristics of immunoregulatory enzymes, it is possible to propose strategies for identifying new members of the group. A fundamental criterion for potential candidates is that enzymes involved in immune regulation must be responsive to immune activation. This feature can be assessed using bioinformatics approaches, such as analyzing the promoter sequence of the gene encoding the protein to identify binding sites for the proteins involved in pro- or anti-inflammatory signaling pathways [52], such as NF-kB [53]. If a protein lacks binding sites for known signaling factors, it may still play a role in immune regulation by being indirectly activated through alternative signaling pathways not yet directly linked to the inflammatory response. In such cases, differential gene expression analysis [54] upon immune activation can be used to identify potential candidates. The most promising candidates should yield positive results in both of these approaches. Once an enzyme’s activation during the immune response is confirmed, its regulatory mechanism is then determined.

**Deprivation of an essential or conditionally essential compound**

A distinctive feature of this mechanism is that suitable properties may be found in enzymes involved in the catabolism of essential compounds. These enzymes may either be the first in the cascade of metabolic reactions (as seen with IDO1 and ARG1) or act as rate-limiting enzymes within the metabolic pathways of the respective substrates. A critical aspect is the identification of essential compounds, since it has been demonstrated that in activated immune cells exhibiting a significantly increased anabolic activity, certain substrates become essential even if they can be synthesized by the body. For instance, glutamine is required for the proliferative response of T-lymphocytes, as shown in human and animal cell cultures [55]. This suggests that glutaminase 1 is a potential immunoregulatory enzyme. In human cell cultures, inhibition of glutaminase 1 was shown to suppress the proliferation of CD4^+^ T-lymphocytes [56], which is consistent with the role of glutaminolysis in supporting lymphocyte proliferation.* Mycobacterium tuberculosis *has recently been found to inhibit glutaminase 1 in murine macrophage cultures, promoting pathogen survival [57]. Tumor cells (as actively proliferating cells) or tumor microenvironment components may also leverage glutaminase to enhance glutamine metabolism, which is associated with a reduced antitumor immune response [58]. However, it remains unknown whether the immune system *per se *employs regulatory mechanisms mediated by glutaminase 1. Specifically, it is unclear whether certain immune cells, by consuming glutamine, can deplete this amino acid and thereby regulate the function of other immune cells, analogous to the mechanism of IDO1.

Vitamins are essential compounds required for the proliferation and differentiation of all cells, including those of the immune system [59]. Therefore, enzymes involved in vitamin metabolism may potentially possess immunoregulatory functions and could be classified as immunoregulatory enzymes. A notable example is dihydrofolate reductase, which is involved in folic acid metabolism. Folic acid deficiency was shown to affect the activity of immune cells in mice [60]. Moreover, experimental studies in mice have demonstrated that targeted depletion of T-lymphocyte populations expressing high levels of the folate receptor can be used to modulate immune responses [61]. In this context, folic acid deficiency within this specific subpopulation of immune cells may lead to functional impairments. However, it remains unknown whether immune cell populations can be regulated through folate depletion *in vivo*.

**Synthesis of a regulatory metabolite**

Many metabolites with signaling functions, such as hormones and neurotransmitters, are potential regulators of immune activity. For example, serotonin was shown to influence the proliferation and cytokine release of various immune cell types [62], making tryptophan hydroxylase a potential immunoregulatory enzyme. Another enzyme with an immunosuppressive function is L-amino acid oxidase (IL4I1), which mediates the synthesis of the tryptophan metabolites that activate AhR, similar to IDO1. This leads to immunosuppression and tumor progression in murine models, although further research is needed to confirm whether IL4I1 is actively utilized by immune cells *per se *[63]. A key feature of the enzyme triad — IDO1, IL4I1, and tryptophan hydroxylase — is their shared substrate, tryptophan. This suggests that a rational approach to identifying potential immunoregulatory enzymes involved in the synthesis of regulatory metabolites is to focus on enzymes that metabolize substrates already utilized by known immunoregulatory enzymes, or those involved in the synthesis of low-molecular-weight hormones and neurotransmitters. For instance, the neurotransmitter gamma-aminobutyric acid (GABA) is synthesized by immune cells and influences their function, making glutamate decarboxylase a potential immunoregulatory enzyme [64].

**Non-enzymatic activity**

A significant number of proteins with multiple biological activities have been identified [65]. The enzymes within this category represent potential immunoregulatory enzymes. Non-enzymatic regulatory activity is not confined to GAPDH but is also observed in

## PROSPECTS FOR THE RESEARCH INTO IMMUNOREGULATORY ENZYMES

The research into immunoregulatory enzymes is not only of fundamental significance, but also holds great potential for medical applications. Technologies leveraging the functions of immunoregulatory enzymes have promising prospects in clinical practice. One of the best studied approaches is the use of immunoregulatory enzyme inhibitors. IDO1 inhibitors have been investigated as immunotherapeutic antitumor agents. Although their efficacy as monotherapy has been limited, these drugs exhibit synergistic capabilities when combined with immune checkpoint inhibitors [68]. Arginase inhibitors are also being explored as potential immunotherapeutic agents for cancer treatment [69]. Another strategy involves direct application of immunoregulatory enzymes. For example, recombinant human arginase has been used as an antitumor agent against arginine-auxotrophic tumors [70]. In murine experiments, the enzyme was injected into tumor tissue alongside standard therapy, utilizing the same essential substrate deprivation principle that underlies the regulation of rapidly proliferating cells. This strategy may be further enhanced by using scaffolds incorporating enzymes or their inhibitors for a localized modulation of the immune function. This approach, which is currently being actively developed for various immunomodulators [71], may have potential applications in cancer immunology, transplantation medicine, and the treatment of infectious and autoimmune diseases.

## CONCLUSIONS

Immunoregulatory enzymes represent a relatively new field of research, and further studies are required for their identification, classification, and mechanistic characterization. By considering the features outlined in this review, the discovery of new members of this group may be made easier, as substantial knowledge already exists about metabolic reactions involving essential compounds and the enzymes induced by pro- or anti-inflammatory cytokines. Such proteins are the most promising candidates in terms of potential immunoregulatory properties. Regulation of immune responses through metabolism enriches our understanding of immune system biology and provides opportunities for the development of novel targeted interventions. The formation of feedback mechanisms through metabolic pathways may be leveraged for therapeutic purposes, allowing immune modulation through the administration of substrates, inhibitors, or enzymes *per se*, depending on the specific context of the disease.

## Abbreviations

IDO1: indoleamine 2,3-dioxygenase 1
ARG1: arginase 1
GAPDH: glyceraldehyde-3-phosphate dehydrogenase
IFN-γ: interferon-gamma
Th1: T helper type 1
AhR: aryl hydrocarbon receptor
Th2: T helper type 2
PBMC: peripheral blood mononuclear cells
iNOS: inducible nitric oxide synthase
NO: nitric oxide
IL-12: interleukin 12
Th17: T helper type 17
ENTPD1: ectonucleoside triphosphohydrolase 1
PAMPs: pathogen-associated molecular patterns
